# Inflammation-Related Gene Signature: An Individualized Risk Prediction Model for Kidney Renal Clear Cell Carcinoma

**DOI:** 10.1155/2022/2559258

**Published:** 2022-01-31

**Authors:** Ze Zhang, Yan-Yan Wei, Qiong-Mei Guo, Chang-Hao Zhou, Nan Li, Jin-Fang Wu, Ya-Ting Li, Wei-Wei Gao, Hui-Li Li

**Affiliations:** ^1^Department of Anesthesiology, The First Hospital of Hebei Medical University, Shijiazhuang, Hebei, China; ^2^Department of Operating Room, The First Hospital of Hebei Medical University, Shijiazhuang, Hebei, China; ^3^Pharmacy Intravenous Admixture Services, The First Hospital of Hebei Medical University, Shijiazhuang, Hebei, China

## Abstract

**Background:**

There is much evidence that confirms the inextricable link between inflammation and malignancy. Inflammation-related regulators were involved in the progression of kidney renal clear cell carcinoma (KIRC). However, the predictive role of single gene biomarkers is inadequate, and more accurate prognostic models are necessary. We undertook the current research to construct a robust inflammation-related gene signature that could stratify patients with KIRC.

**Methods:**

The transcriptome sequencing data along with clinicopathologic information of KIRC were obtained from TCGA. A list of inflammation-related genes was acquired from the Molecular Signatures Database. Using the RNA-seq and survival time data from the TCGA training cohort, an inflammation-related gene signature was built using bioinformatic methods, and its performance in predicting patient prognosis was assessed by Kaplan–Meier and ROC curve analyses. Furthermore, we explored the association of risk score with immune score, stromal score, tumor immune-infiltrating cells (TIICs), immunosuppressive molecules, m6A regulators, and autophagy-related biomarkers.

**Results:**

Herein, nine inflammation-related hub genes (ROS1, PLAUR, ACVR2A, KLF6, GABBR1, APLNR, SPHK1, PDPN, and ADORA2B) were determined and used to build a predictive model. All sets, including training set, four testing sets, and the entire TCGA group, were divided into two groups (low and high risk), and Kaplan–Meier curves all showed an adverse prognosis for patients in the high-risk group. ESTIMATE algorithm revealed a higher immune score in the high-risk subgroup. CIBERSORT algorithm illustrated that the high-risk group showed higher-level immune infiltrates. Furthermore, LAG3, TIGIT, and CTLA4 were overexpressed in the high-risk subgroup and positively associated with risk scores. Moreover, except for METTL3 and ALKBH5, the other m6A regulators decreased in the high-risk subgroup.

**Conclusions:**

In conclusion, a novel inflammation-related gene signature comprehensively constructed in the current study may help stratify patients with KIRC.

## 1. Introduction

Kidney renal clear cell carcinoma (KIRC) is the most lethal urological tumor and its incidence and mortality are increasing yearly [1]. Radical surgery is the preferred treatment of limited renal clear cell carcinoma. Then, 20–40% of patients in the early stages eventually develop metastatic KIRC. Moreover, approximately 30% of patients with renal clear cell carcinoma have a metastasis initial diagnosis due to insidious onset [2]. Unlike other advanced malignancies, advanced renal clear cell carcinoma is resistant to conventional radiotherapy, and although the advent of targeted drugs such as tyrosine kinase and mTOR pathway inhibitors has enhanced the long-term survivals for several patients, the clinical outcome for most patients remains poor due to the presence of toxic side effects and the emergence of drug resistance [3, 4].

The link between cancer and inflammation has been explored extensively since it was discovered in the 19th century. Several lines of evidence suggest that tumors usually occur in the site of chronic inflammation and inflammatory cells exist in the biopsy of tumor [5]. Researchers found that inflammation mediators and cellular effects are essential components of the local tumor environment [6]. In several types of cancer, inflammation exists prior to the development of malignant changes. In contrast, carcinogenic changes in other types of cancer can induce an inflammatory microenvironment and promote tumor progress [7]. Whatever its origin, the inflammation in the tumor microenvironment has many tumorigenesis effects. It not only accelerates tumor progression by promoting the proliferation, angiogenesis, and metastasis, but also disrupts adaptive immune responses and makes tumor cells tolerant to hormones and chemotherapy drugs. This cancer-related inflammatory molecular pathway is now being uncovered [8]. Balkwill et al. [9] have revealed that the invasion ability of neoplastic cells is increased in the presence of inflammatory cytokines. Tan et al. [10] have shown that inflammation-related genes might serve as important prognostic biomarkers for assessing recurrence risk (GADD45G) and death (CARD9, CIITA, and NCF2) in patients with KIRC. At present, some therapeutic drugs for inflammatory cytokines are being developed and tested in clinical practice [11], suggesting that targeting inflammation-related genes is a promising cancer therapy.

As mentioned above, targeting inflammation-related biomarkers may be a promising novel choice for tumor treatment. A large number of inflammation-related regulators are associated with the KIRC progression; however, cancer is a disease caused by the combined involvement of multiple genes and pathways. Given the limitations of a single biomarker, we screened multiple inflammation-related genes for prognostic relevance and constructed a gene signature for risk stratification and prognostic assessment of patients. Herein, we aim to develop an inflammation-related lncRNA model to predict the survival outcomes of patient with KIRC. We used the TCGA database to develop and validated the individualized prognostic signature for KIRC based on inflammation-related genes. Combined with the inflammation-related genes with clinical variables, we construct a comprehensive gene model that could assess the prognosis of patients with KIRC.

## 2. Materials and Methods

### 2.1. Data Collection

RNA-Seq gene expression data for KIRC was downloaded from the TCGA database (https://portal.gdc.cancer.gov/), called TCGA-KIRC. The reads per map per million base pairs (FPKM) counts and fragment counts per thousand transcripts were downloaded for further analysis. We finally obtained RNA sequencing data from 530 patients with complete clinical information and their clinicopathological data.

### 2.2. Identification of Differentially Expressed Inflammation-Related Genes (DE-IFRGs)

A comprehensive list of inflammation-related genes (IFRGs) was retrieved from the hallmark gene sets from the Molecular Signatures Database v7.4 (http://www.gsea-msigdb.org/gsea/msigdb/index.jsp), which consists of 200 IFRGs. The “limma” *R* package and the Wilcoxon test method were used to identify the DE-IFRGs with an adjusted *P* < 0.05 between KIRC and adjacent normal renal tissues. The “pheatmap” *R* package was employed to visualize the degree range of differences in the TCGA-KIRC datasets.

### 2.3. Gene Ontology (GO) and Kyoto Encyclopedia of Genes and Genomes (KEGG)

To reveal the potential biological functions and underlying action mechanisms of DE-IFRGs, we conducted the GO and KEGG analyses applying the “clusterProfiler” *R* package [12]. Functional enrichment items were considered as “functional” when the false discovery rate (FDR) <0.05.

### 2.4. Building and Verifying a Prognostic Inflammation-Related Gene Signature

According to the ratio of 6 : 1 : 1 : 1 : 1, all patients were randomly randomized into five cohorts, including training set (*n* = 320), testing-1 set (*n* = 53), testing-2 set (*n* = 52), testing-3 (*n* = 52), and testing-4 set (*n* = 53). Firstly, using the data from the training set, prognosis-related DE-IFRGs were selected by the univariate Cox analysis (*P* < 0.001). Then, we further reduce the amount of genes using the LASSO regression analysis to prevent overfitting. Finally, multivariate assays were conducted to identify the hub IFRGs and build a prognostic signature. We then calculate the risk score for each KIRC patient using the following formula: exp gene 1 ^*∗*^*β* gene 1 + exp gene 2 ^*∗*^*β* gene 2 + exp gene 3 ^*∗*^*β* gene 3 + … exp gene *n*^*∗*^*β* gene *n*. Furthermore, patients in all sets as well as the entire TCGA set were classified into low- and high-risk subgroups according to the median risk score of the training set. Then, survival assays were conducted. ROC assays were utilized to measure the predictive capability of the prognostic model.

### 2.5. Evaluation of the Risk Signature

Uni- and multivariate Cox regression analyses were conducted to select the independent prognostic factors. Besides, the associations between risk scores and clinical features of patients were studied. Then, we construct a nomogram consisting of independent prognostic factors to predict the OS of KIRC patients. Calibration curve was employed to compare the differences between predicted OS and actual OS. In addition, we compared the differences in the ability of risk model as well as clinicopathological variables to assess patient prognosis.

### 2.6. Functional Enrichment Analysis

Differentially expressed genes (DEGs) between the high- and low-risk subgroups were identified using the “limma” *R* package. Genes with |log2FC| ≥ 1, FDR <0.05 were considered differentially expressed. Then, GO and KEGG assays based on these DEGs were carried out applying the “clusterProfiler” *R* package [12].

### 2.7. Evaluation of the Tumor Microenvironment (TME) and Tumor Infiltrated Immune Cells (TIICs)

The ESTIMATE algorithm was used to evaluate scores representative of the relative proportion of immune and stromal cells. Furthermore, we further compared the difference of immune and stromal scores between high- and low-risk subgroups by the Wilcoxon test. Additionally, to analyze the relationships between risk score and TIICs, the content of TIICs was calculated using the CIBERSORT algorithm (http://cibersort.stanford.edu/).

### 2.8. Association of Risk Score with Immunosuppressive Molecule, m6A Regulators, and Autophagy-Related Biomarkers

Considering immune checkpoint inhibitors (ICIs) were clinically employed to treat KIRC, we evaluated the association between risk score with ICI-related regulators. m6A regulators and autophagy-related biomarkers were closely related to cancer progression; we thus evaluated the correlation between risk score and m6A regulators as well as autophagy-related biomarkers.

### 2.9. Statistical Analysis

All statistical analyses were carried out using *R* (version 3.6.1). Univariate, LASSO, and multivariate assays were used to select the prognostic genes and develop a gene signature. The Kaplan–Meier analysis was applied to show the survival difference. ROC assays were applied to estimate the predictive performance of the risk model. The independent prognostic factors were determined applying multivariate assays. Wilcoxon's test and Pearson's correlation methods were utilized to evaluate the association of risk score with TME, TIICs, ICI-related regulators, m6A regulators, and autophagy-related biomarkers. *P*-value <0.05 was considered statistically significant.

## 3. Results

### 3.1. Data Preparation

**Figure 1:**
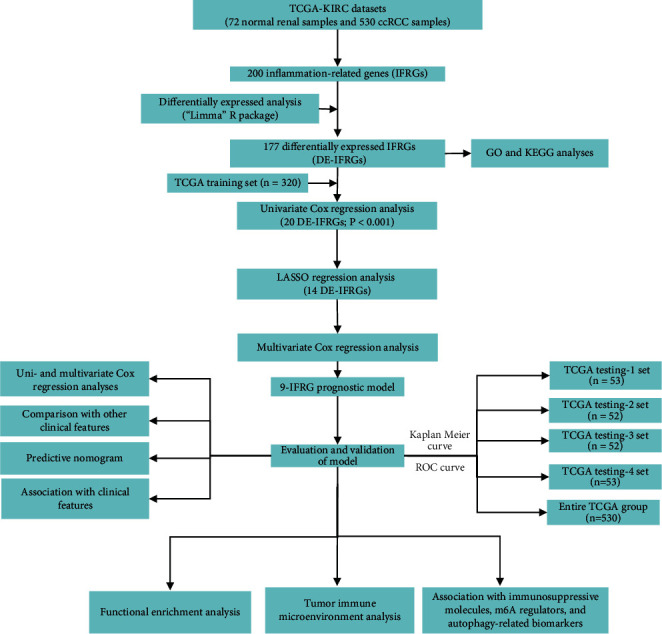
The flowchart describes the gene signature of KIRC established in this study and its comprehensive analysis.

The detailed workflow flowchart of this study is listed in Figure 1. The transcriptome profiles and clinical information of 530 patients with KIRC were publicly downloaded from the TCGA database. We then randomly divided all patients into the training set (*n* = 320), testing-1 set (*n* = 53), testing-2 set (*n* = 52), testing-3 (*n* = 52), and testing-4 set (*n* = 53). Data from the training set was used to choose prognosis-related hub IFRGs and construct a risk signature. Simultaneously, data from testing-1, testing-2, testing-3, and testing-4 sets as well as the entire group was utilized to demonstrate the capability of the risk score.

### 3.2. Identification of DE-IFRGs

**Figure 2:**
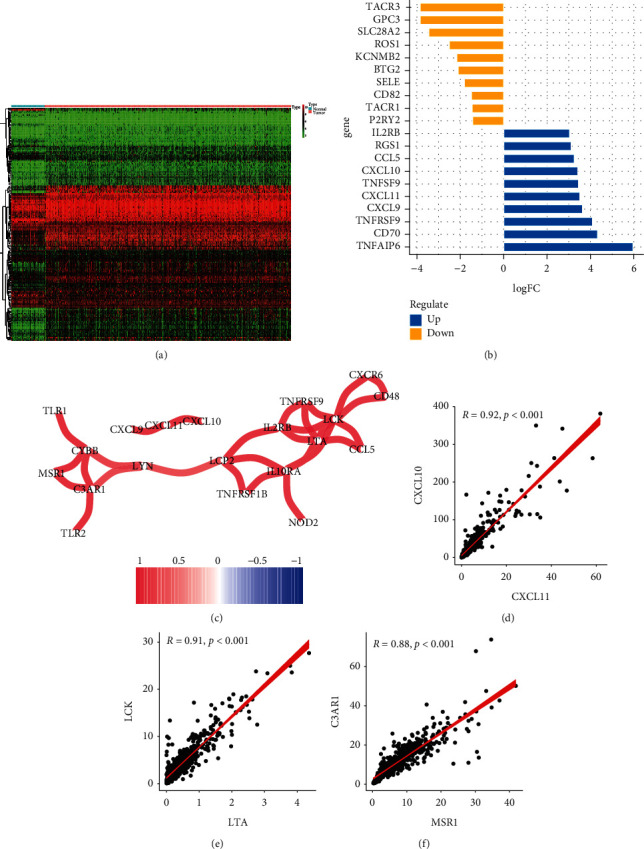
Identification of differentially expressed inflammation-related genes (DE-IFRGs) between normal tissues and KIRC tissues. (a) The heat map of DE-IFRGs. (b) The top ten upregulated and downregulated PRGs. (c) Correlation network between DE-IFRGs (Pearson's coefficient >0.8). (d–f) The strongest correlations were found between CXCL11 and CXCL10, LTA and LCK, and MSR1 and C3AR1.

The “limma” *R* package was employed to screen the differentially expressed DE-IFRGs between KIRC samples and normal renal specimens. Herein, 177 dysregulated genes were identified, of which 46 were downregulated, and 131 were upregulated (Figure 2(a)).  Figure 2(b) shows the top ten up- and downregulated IFRGs in KIRC. Additionally, we calculate the Pearson coefficients DE-IFRGs, and Figure 2(c) showed a strongly correlated DE-IFRGs association map (cor > 0.8 and *P* < 0.05), of which the strongest correlations were found between CXCL11 and CXCL10, LTA and LCK, and MSR1 and C3AR1 (Figures 2(d)–2(f)).

### 3.3. Functional Enrichment Analysis of DE-IFRGs

**Figure 3:**
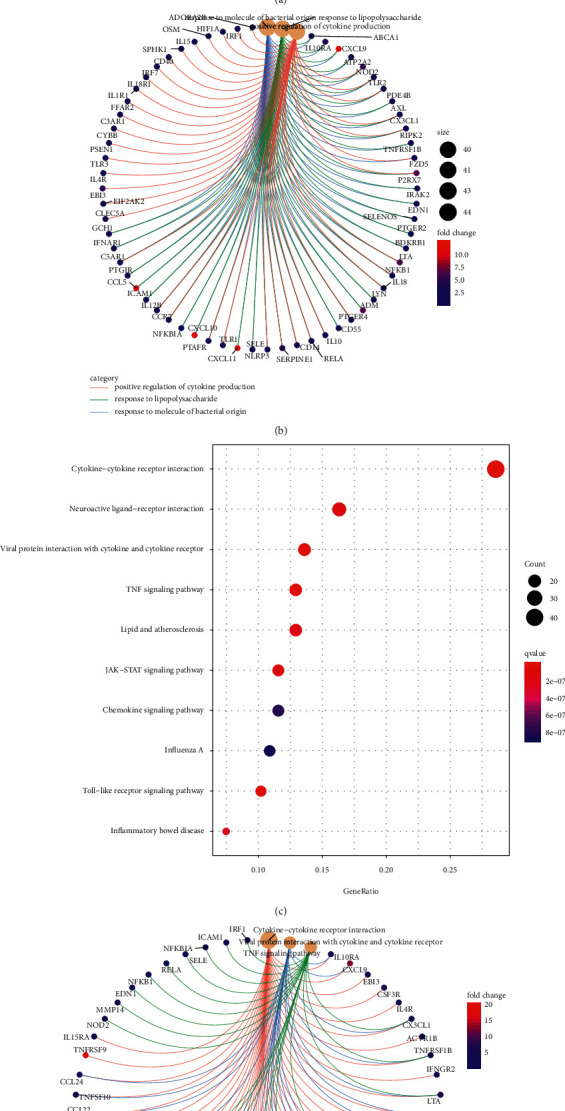
Functional enrichment analysis of DE-IFRGs. (a) GO enrichment analysis of DE-IFRGs. (b) Enriched GO enrichment terms and corresponding DE-IFRGs. (c) KEGG signaling pathway analysis of DE-IFRGs. (d) Enriched cancer-related pathways and corresponding DE-IFRGs.

Functional enrichment analysis of these DE-IFRGs was conducted using the “clusterProfiler” *R* package. As revealed in Figure 3(a), the significantly enriched BP terms were response to molecule of bacterial origin, response to lipopolysaccharide, and positive regulation of cytokine production; in terms of CC, DE-IFRGs were mainly involved in positive regulation of cytokine production, secretory granule membrane, and membrane raft; as for MF, DE-IFRGs were mainly involved in receptor-ligand activity, cytokine receptor binding, and cytokine activity.  Figure 3(b) showed the three significantly enriched GO terms and relevant DE-IFRGs involved in them. Additionally, the top 10 KEGG pathways were TNF signaling, lipid and atherosclerosis, JAK-STAT signaling, chemokine signaling pathway, Influenza A, Toll-like receptor signaling pathway, and inflammatory bowel disease (Figure 3(c)).  Figure 3(d) displays the three significantly enriched signaling pathways and related DE-IFRGs involved in these pathways.

### 3.4. Construction and Validation of a Risk Signature Based on Prognosis-Related IFRGs

**Figure 4:**
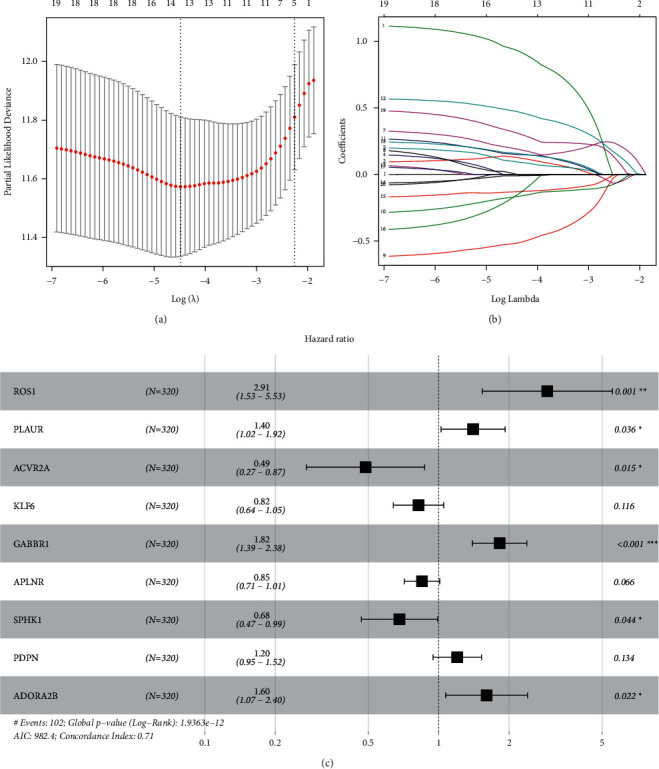
Identification of a 9-gene risk signature for overall survival by multivariate Cox regression analysis. (a) The minimum number corresponds to the covariates. (b) The changing trajectory of each independent variable. (c) Nine prognosis-associated hub IFRGs were identified by further multivariate Cox regression analysis.

**Figure 5:**
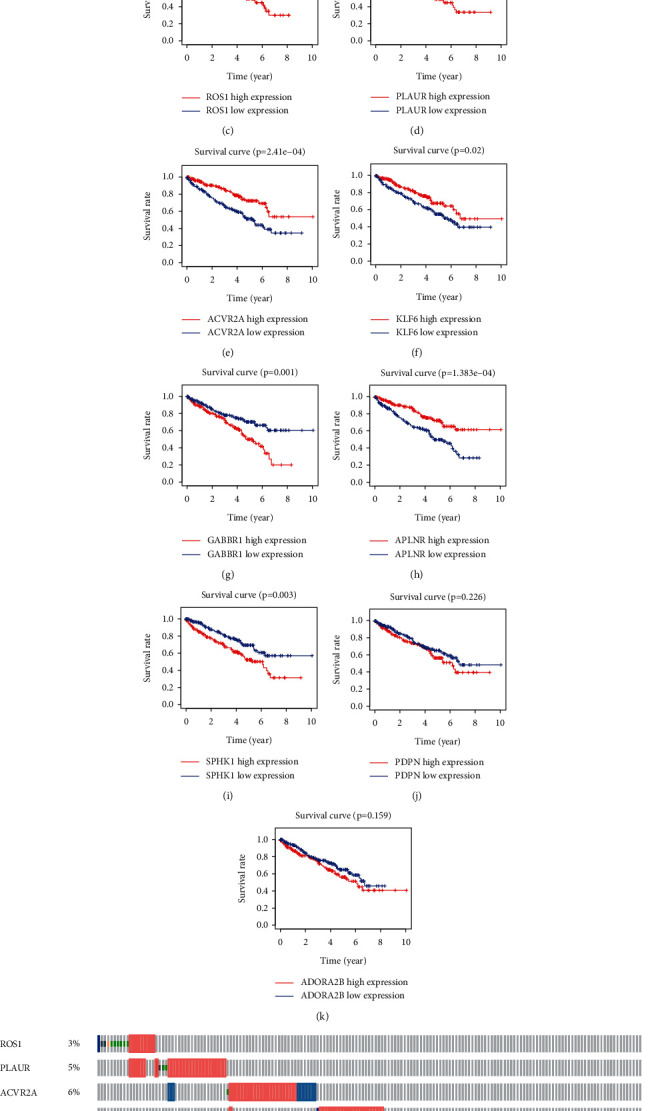
Comprehensive analysis of nine prognosis-associated hub IFRGs. (a) Heat map of expression of nine hub IFRGs between high and low risk subgroups (^*∗∗∗*^*P* < 0.001). (b) Differential expression of nine hub IFRGs between high- and low-risk subgroups. (c–k) The Kaplan–Meier curves of ROS1, PLAUR, ACVR2A, KLF6, GABBR1, APLNR, SPHK1, PDPN, and ADORA2B, respectively. (l) Genetic alteration of nine hub IFRGs in KIRC patients.

**Figure 6:**
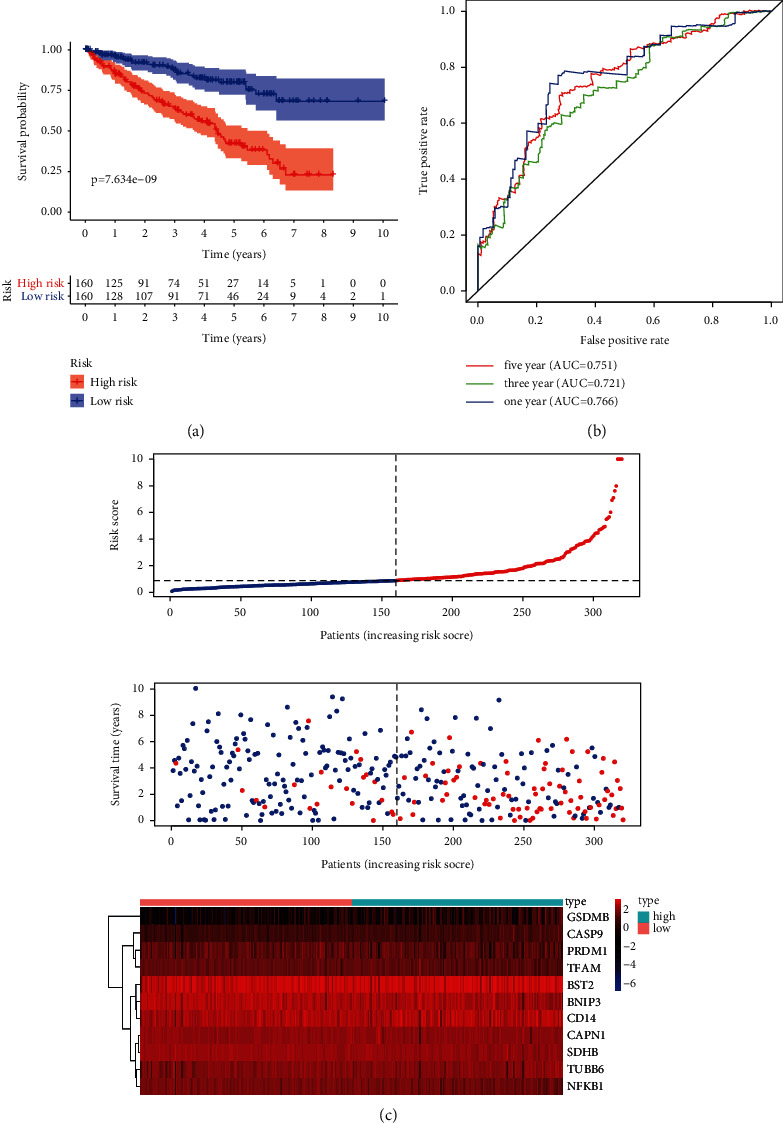
Development of the prognostic signature based on nine hub IFRGs. (a) Survival curve for low- and high-risk subgroups in the TCGA training cohort. (b) Time-dependent ROC curve of the 9-IFRG prognostic risk signature. (c) Relationships among the risk score (upper), survival status of patients (middle), and the expressing pattern of the genes (bottom).

**Figure 7:**
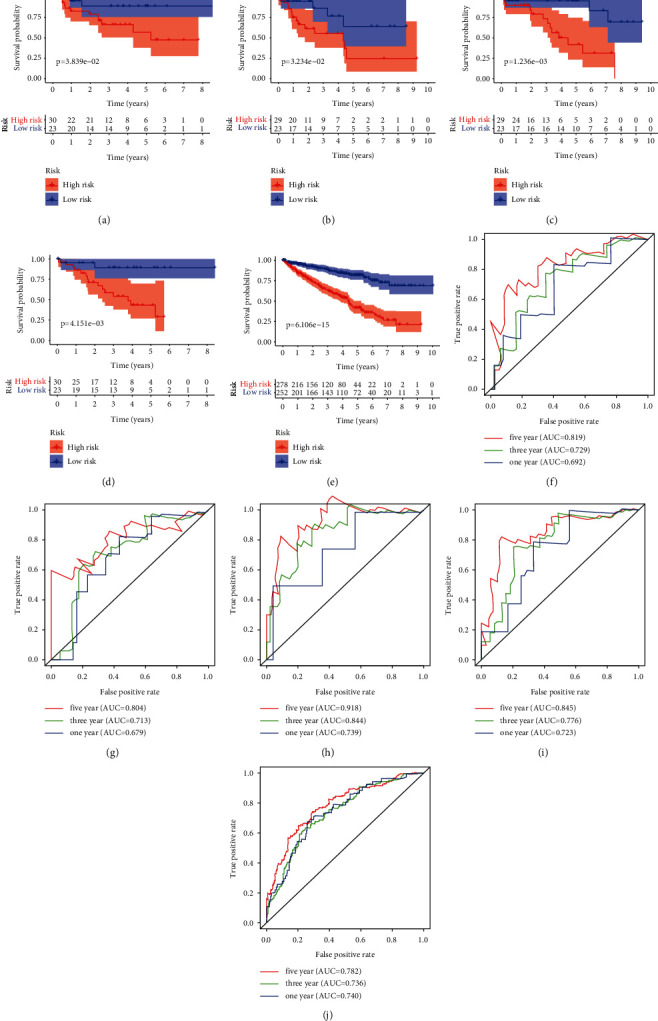
Validation of the prognostic signature based on nine inflammation-related genes in different cohorts. (a–e) Survival assays of the 9-IFRG prognostic signature in the testing-1 cohort, testing-2 cohort, testing-3 cohort, testing-4 cohort, and the entire group, respectively. (f–j) Time-dependent ROC curves of the 9-IFRG prognostic risk signature in the four cohorts.

Using univariate Cox regression analysis, 20 prognosis-related IFRGs were identified (*P* < 0.001) (Table 1). Subsequently, the least absolute Lasso regression analysis was employed to prevent the overfitting and determine the most important prognosis-related IFRGs in KIRC (Figures 4(a) and 4(b)). Then, stepwise multivariate assays were applied to build a gene signature. Eventually, nine hub IFRGs (ROS1, PLAUR, ACVR2A, KLF6, GABBR1, APLNR, SPHK1, PDPN, and ADORA2B) were used to construct the gene signature (Figure 4(c)). Based on regression coefficients (Table 2), we calculated the risk score for each patient using the following formula: risk score = (1.069 ^*∗*^ ROS1) + (0.339 ^*∗*^ PLAUR) + (−0.720 ^*∗*^ ACVR2A) + (−0.198 ^*∗*^ KLF6) + (0.600 ^*∗*^ GABBR1) + (−0.164 ^*∗*^ APLNR) + (−0.386 ^*∗*^ SPHK1) + (0.183 ^*∗*^ PDPN) + (0.472 ^*∗*^ ADORA2B). As exhibited in Figures 5(a) and 5(b), ROS1, PLAUR, GABBR1, SPHK1, and PDPN were overexpressed in the high-risk subgroup, whereas ACVR2A, KLF6, and APLNR were distinctly decreased in the high-risk subgroup. However, no difference was found in ADORA2B. Moreover, our group observed that overexpression of ROS1 and PLAUR indicated worse overall survival (Figures 5(c) and 5(d)). The downregulation of ACVR2A and KLF6 predicted a poor prognosis of patients (Figures 5(e) and 5(f)). Increased expression of GABBR1 was associated with a shorter OS (Figure 5(g)). Low APLNR expression predicted a shorter OS (Figure 5(h)). Increased expression of SPHK1 and PDPN suggested worse prognosis (Figures 5(i) and 5(j)). Also, no difference was found in ADORA2B (Figure 5(k)). Furthermore, using the cBioPortal database, we explored the genetic mutations of 9 hub IFRGs, and results were shown in Figure 5(l). Subsequently, 320 patients in the training set were stratified into the low- and high-risk subgroups based on the median risk score value. Kaplan–Meier curves showed that high-risk patients showed poorer OS by comparison with low-risk patients (*P* < 0.001) (Figure 6(a)). ROC assays were utilized to evaluate the prognostic performance of the gene signature, and results showed that the area under the ROC curve for 1-year, 3-year, and 5-year OS was 0.766, 0.721, and 0.751 (Figure 6(b)). The survival status and the expressions of 9-IFRGs in the training cohort were presented in −. To verify the predictive performance of the gene model, patients in the testing-1 cohort, testing-2 cohort, testing-3 cohort, testing-4 cohort, and the entire group were classified as high- and low-risk subgroups. The Kaplan–Meier survival curve showed a significantly good OS in the low-risk group (Figures 7(a)–7(e)). The AUC of the gene signature in the testing-1 cohort for 1-year, 3-year, and 5-year OS is also shown in Figures 7(f)–7(j).

### 3.5. Independent Prognostic Analysis, Correlation of Risk Score with Clinical Features, and Construction of a Nomogram

**Figure 8:**
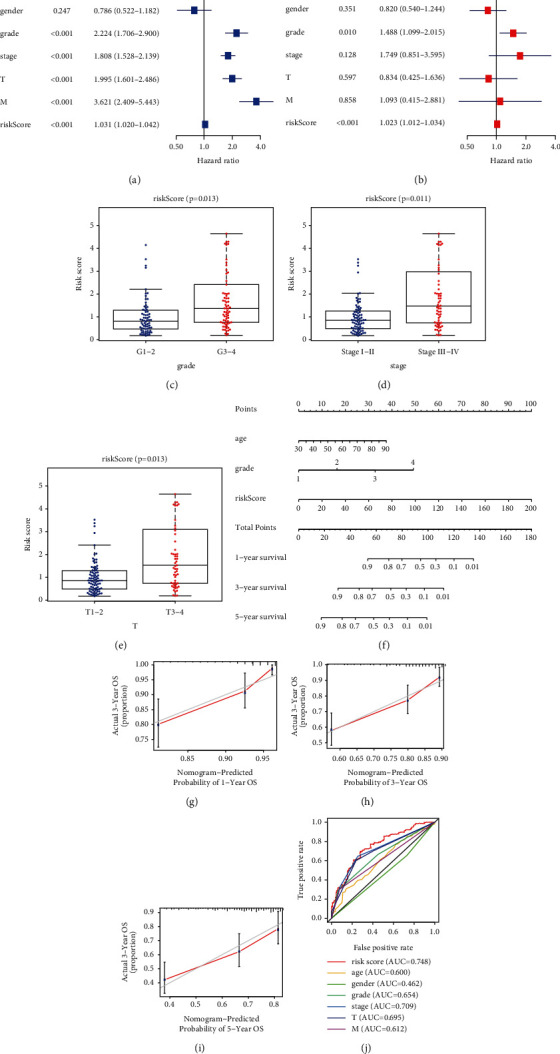
Independent prognostic analysis and construction of nomogram. (a) Univariate Cox regression assays were used to explore the prognostic value of risk score and other clinical features in KIRC. (b) Multivariate Cox regression assays were applied to demonstrate whether risk score and other clinical features could be an independent marker for KIRC patients. (c–e) An elevated risk score was significantly correlated with higher histological grade, advanced clinical stage, and T stage. (f) A nomogram consisting of independent clinical features for predicting 1-, 3-, and 5-year OS of KIRC. (g–i) Calibration curves of 1-year, 3-year, and 5-year OS of KIRC. (j) ROC curves for the superiority of the gene signature.

By coupling with the risk model and clinicopathological features, we identified the risk score (HR = 1.023, *P* < 0.001) as a factor of overall survival for KIRC using uni- and multivariate Cox regression analyses (Figures 8(a) and 8(b)). Besides, we showed that elevated risk score was notably correlated with higher histological grade (*P* < 0.05, Figure 8(c)), advanced clinical stage (*P* < 0.05, Figure 8(d)), and T stage (*P* < 0.05, Figure 8(e)), suggesting that risk score was positively correlated with tumor progression. Moreover, we used the independent prognostic factors to establish a prognostic nomogram (Figure 8(f)), and calibration curves showed that the nomogram performed well at predicting 1-, 3-, and 5-year OS in KIRC patients (Figures 8(g)–8(i)), indicating the robust predictive ability of the prognostic nomogram. Additionally, we found that risk score had the largest AUC compared with other clinical variables in predicting 5-year OS of KIRC (Figure 8(j)), suggesting that risk score has advantages over other clinical traits in estimating 5-year OS of KIRC.

### 3.6. Functional Enrichment Analyses

**Figure 9:**
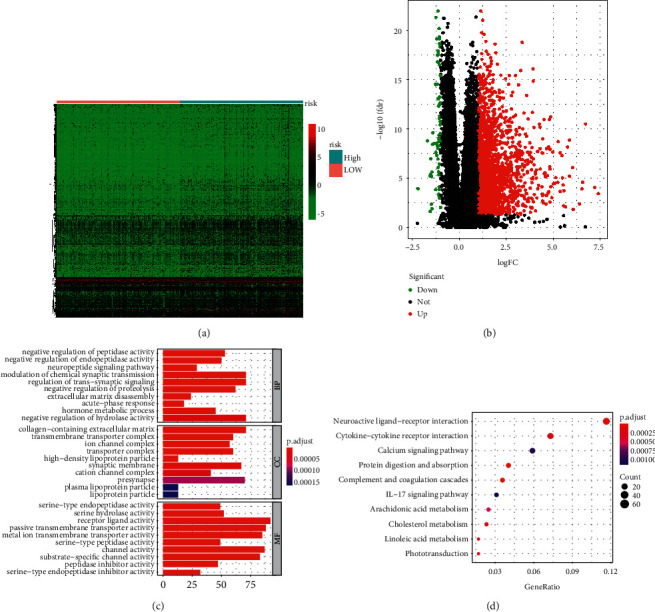
Functional assays of DEGs between high- and low-risk subgroups. (a) Heat map of DEGs between high- and low-risk subgroups. (b) Volcano map of DEGs between high- and low-risk subgroups. (c) Significantly enriched GO enrichment terms. (d) KEGG signaling pathway analysis.

To illustrate the underlying action mechanisms related with the 9-IFRG signature-derived risk model, a total of 1,771 DEGs were identified between high- and low-risk subgroups. Figures 9(a) and 9(b) shows the heat map and volcano map of DEGs, respectively. As illustrated in Figure 9(c), concerning biological processes, DEGs were significantly enriched in the modulation of negative regulation of hydrolase activity; with regard to cellular components, DEGs were significantly involved in the collagen-containing extracellular matrix, presynapse, and synaptic membrane; in point of molecular functions, DEGs were noticeably involved in receptor-ligand activity, passive transmembrane transporter activity, and channel activity. DEGs were mainly enriched in phototransduction, linoleic acid metabolism, cholesterol metabolism, arachidonic acid metabolism, IL-17 signaling pathway, and protein digestion and absorption (Figure 9(d)).

### 3.7. Association of Risk Score with TME

**Figure 10:**
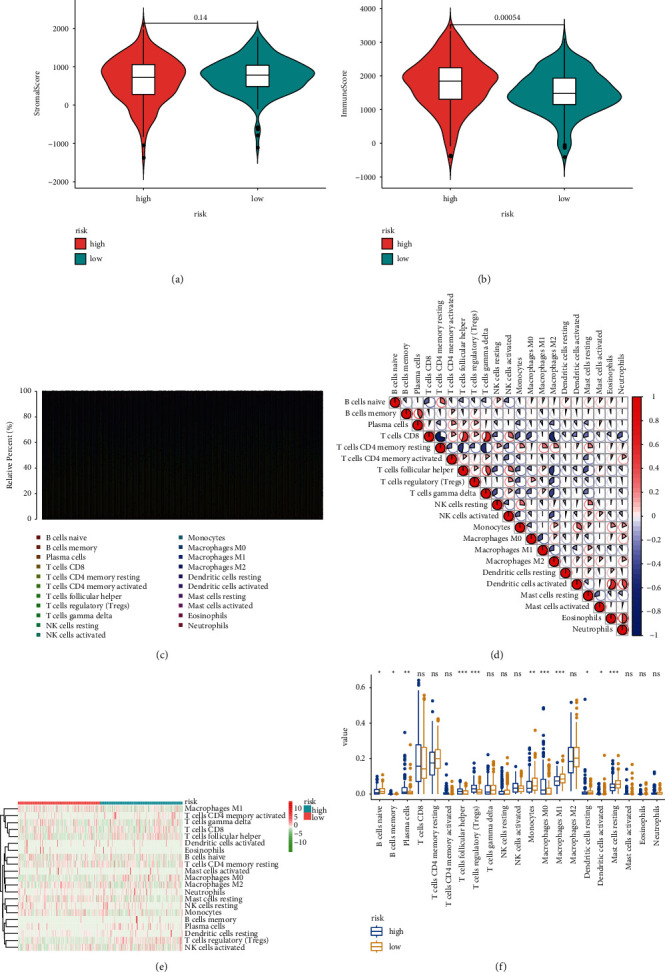
Association of risk score with tumor immune microenvironment in KIRC. (a) Differences in stromal score between high- and low-risk subgroups. (b) Differences in immune score between high- and low-risk subgroups. (c) Relative proportion of immune infiltration in KIRC. (d) Correlation between tumor-infiltrating immune cells. (e) The heatmap exhibited the infiltrating difference of immune cells in two groups. (f) Barplot showed the ratio differentiation of 21 kinds of immune cells.

Immune and stromal cells are crucial constituents of the immune microenvironment. In this current study, the contributions of stromal and immune cells to KIRC were estimated by the ESTIMATE algorithm. The results signified that immune score was crucially higher in the high-risk group (Figure 10(a)); however, no difference was found for the stromal score (Figure 10(b)). Additionally, we applied the CIBERSORT algorithm to compare the differences in each type of immune infiltrating cells.  Figure 10(c) showed the proportion of 21 immune cells in each sample.  Figure 10(d) illustrates the correlations between infiltrated immune cells in the tumor.  Figure 10(e) shows the heat map of the 21 immune cell proportions. Moreover, Figure 10(f) shows the relationship between risk score with different immune cells, and we found that the high-risk group showed higher-level immune infiltrates of M0 macrophages, regulatory T cells (Tregs), follicular helper T cells, plasma cells, and memory B cells.

### 3.8. Association of Risk Score with Immunosuppressive Molecules, m6A Regulators, and Autophagy-Related Biomarkers

**Figure 11:**
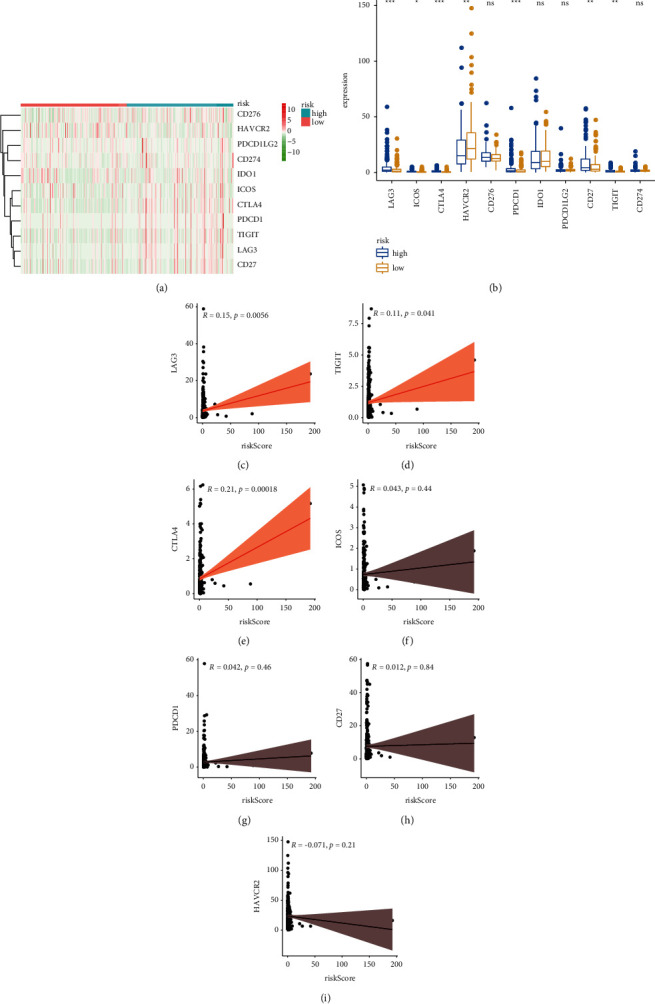
Differences in immunosuppressive molecule expression between high- and low-risk subgroups. (a) The heatmap exhibited the immunosuppressive molecules in two groups. (b) Barplot showed the immunosuppressive molecules between KIRC specimens with low- or high-risk subgroups relative to the median of risk score. (c–i) Association of LAG-3, TIGIT, CTLA4, ICOS, PDCD1, CD27, and HAVCR2 with the risk score.

**Figure 12:**
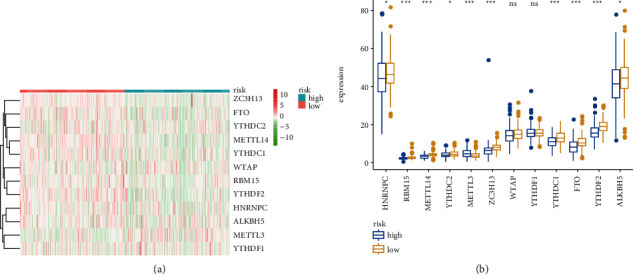
Association of risk score with m6A regulators in KIRC. (a) The heatmap exhibited the immunosuppressive molecules in two groups. (b) Barplot showed the m6A regulators between KIRC specimens with low- or high-risk subgroups.

**Figure 13:**
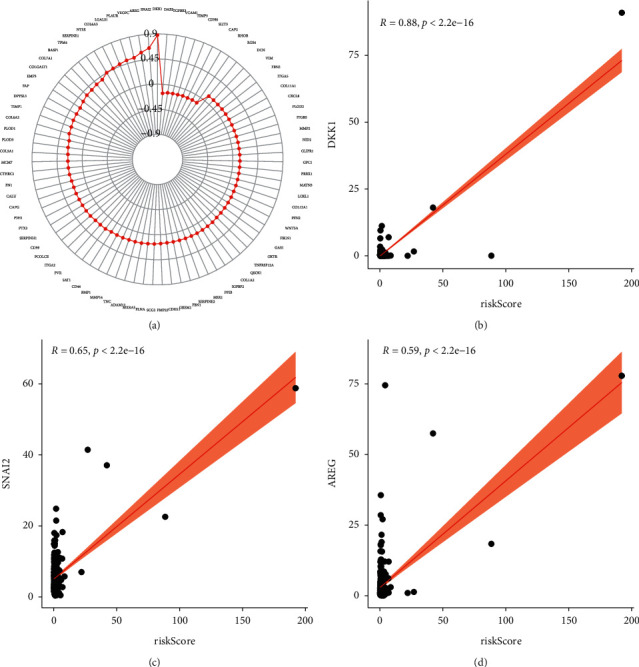
Association of risk score with autophagy-related genes in KIRC. (a) Autophagy-related genes significantly associated with risk score. (b–d) The top three autophagy-related genes associated with risk score are DKK1, SNAI2, and AREG.

Then, we estimated the association between immunosuppressive molecules and risk score.  Figure 11(a) shows the heat map of common immunosuppressive molecules in high- and low-risk subgroups. Furthermore, as illustrated in Figure 11(b), patients with high-risk score expressed higher levels of LAG-3, ICOS, CTLA4, PDCD1, CD27, and TIGIT, whereas HAVCR2 was overexpressed in patients with the low-risk score. Correlation analysis confirmed that LAG-3 (cor = 0.15, Figure 11(c)), TIGIT (cor = 0.11, Figure 11(d)), and CTLA4 (cor = 0.21, Figure 11(e)) were positively associated with the risk score, whereas no difference was found for ICOS, PDCD1, CD27, and HAVCR2 (Figures 11(f)–11(i)). Together, these results indicate that LAG-3, TIGIT, and CTLA4 were positively associated with the risk score. Recent evidence indicated the vital role of m6A mRNA methylation in reducing the antitumor response of CD8 + T cells and promoting anti-PD-1 drug resistance. We thus assess the relationship between risk score and m6A regulators.  Figure 12(a) shows the heat map of common m6A regulators in high- and low-risk subgroups. Additionally, we discovered that most of the m6A regulators were significantly decreased in the high-risk subgroup except for METTL3 (Figure 12(b)). The results indicate that high-risk subgroup patients may be more suitable for immunotherapy with emerging checkpoint inhibitors. Growing researches have revealed a key role for autophagic pathways and proteins in immunity and inflammation. We thus explore the association of autophagy-related genes with risk score, and we found that several autophagy-related genes have a significant link with risk score (Figure 13(a)), and the top three relevant autophagy-related genes are DKK1 (Figure 13(b)), SNAI2 (Figure 13(c)), and AREG (Figure 13(d)).

## 4. Discussion

In this work, we constructed an inflammation-related gene feature and evaluated its predictive capability in predicting OS of KIRC patients. Then, we studied the potential functions and signaling pathways closely related to risk score and further explored the association between risk score with immune microenvironment, immunosuppressive molecules, m6A regulators, and autophagy-related biomarkers. Here, nine hub IFRGs (ROS1, PLAUR, ACVR2A, KLF6, GABBR1, APLNR, SPHK1, PDPN, and ADORA2B) were selected by bioinformatics and used to construct 9-IFRG risk signature successfully. Afterwards, we found that gene signature performed well in the training set, testing-1 set, testing-2 set, testing-3 set, testing-4, and the entire TCGA group. Specifically, the higher the risk score of patients is, the worse the overall survival rate is. ROC curve also confirms the robust predictive performance of the risk model. Additionally, by combining the risk model with the clinicopathological features of patients, we found that the 9-IFRG gene model can independently predict the OS of patients with KIRC. Further investigation indicated that the nomogram performed well at predicting 1-, 3-, and 5-year OS in KIRC patients. Furthermore, we found that the risk score was significantly associated with cancer progression in KIRC patients. Moreover, compared to other clinical variables, the risk score had the highest predictive performance of prognosis. To sum up, we constructed a powerful 9-IFRG risk signature and an effective nomogram for KIRC risk stratification and overall survival prediction.

Of the nine hub IFRGs (ROS1, PLAUR, ACVR2A, KLF6, GABBR1, APLNR, SPHK1, PDPN, and ADORA2B) we identified, some are associated with cancer progression. The protooncogene ROS1 encodes a tyrosine kinase receptor that has an essential physiological role in humans. Studies have shown that somatic chromosomal fusions involving ROS1 generate chimerical tumor proteins that can cause various cancers [13]. In inflammatory myofibroblastic tumors, ROS1 expression predicts ROS1 gene rearrangement [14]. PLAUR, also known as u-PAR, is an essential molecule in modulating cell surface fibrinogen activation and plays a vital role in many healthy and pathological processes [15]. Abnormal PLAUR disorders played a key role in the progression and metastasis of human colon cancer [16]. Moreover, PLAUR impacted colorectal liver metastases by influencing the protein hydrolytic activity and inflammation of the tumor microenvironment in colorectal cancer. Consequently, the colorectal liver metastases [17] ACVR2A is a ligand for activin A protein and is closely associated with polyarthrosis syndrome, protointestinal embryogenesis, and spermatogenesis [18]. Emerging evidence indicated that ACVR2A is involved in many cancer-related signaling pathways, such as the PEDF-induced signaling, the TFG-*β* signaling, or signaling pathways regulating stem cell pluripotency [19]. KLF6 is a transcription factor of the zinc finger family and modulates lipid homeostasis in KIRC [20]. Additionally, KLF6 had been found to promote the expression and function of proinflammatory genes by inhibiting miR-223 expression in macrophages [21]. GABBR1, also known as GABABR1, is a 7-transmembrane receptor. In colorectal cancer, decreased GABBR1 fosters the proliferation and invasion; overexpression of GABBR1 has the opposite [22]. APLNR is also a seven-transmembrane G protein-coupled receptor that is universally present in diverse tissues. In osteosarcoma, elevated APLNR expression promotes proliferation and invasion [23]. SPHK1 is a biologically active metabolite of sphingosine that is involved in various tumor progression by enhancing cell proliferation and motility. Currently, drugs targeting SPHK1 are now being progressively validated in clinical trials [24]. Type I integral membrane glycoprotein encoded by PDPN is widely distributed in human tissues. In breast tumor-infiltrating immune cells, PDPN was found highly expressed in tumor-associated macrophages (TAMs), and the latter spurs local stromal remodeling and promotes vascular growth and lymphatic infiltration [25]. ADORA2B is a member of the G protein-coupled receptor superfamily and encodes an adenosine receptor. A recent report indicates that hypoxia-inducible factor 1-dependent expression of ADORA2B facilitates breast cancer stem cell enrichment [26]. The above reports confirmed the role of 9 hub IFRGs in carcinogenesis. However, whether ROS1, PLAUR, ACVR2A, KLF6, GABBR1, APLNR, SPHK1, PDPN, and ADORA2B affect the clinical outcome of KIRC patients via modulating the process of inflammation requires to be further elaborated, and there are few relevant studies.

To elucidate the functional roles associated with the risk score, the DEGs between the high-risk and low-risk subgroups were identified and used to perform functional enrichment analysis. Intriguingly, we noticed that DEGs are involved in several tumor-related signaling pathways. These signaling pathways are all in connection with the regulation of tumor immunity. Through the interaction between chemokines or cytokines and their receptors, different subsets of immune cells are recruited into the tumor microenvironment, causing these populations having a differential impact on tumor progression and treatment outcome [27]. In gastric cancer, elevated intratumoral mast cells resulted in immune suppression via modulating TNF-*α*-PD-L1 pathway [28]. The JAK-STAT signaling pathway is involved in tumor cell recognition and tumor-driven immune escape and plays a role in almost all immune regulatory processes [29]. Toll-like receptor signaling pathway is a classical immune signaling pathway that plays an irreplaceable role in modulating tumor immunity and cancer progression [30]. In addition, we found that the high-risk group had a higher immune score. With regard to immune infiltrating cells, we found that high-risk group showed higher level immune infiltrates. Among them, regulatory T cells (Tregs) play crucial roles in keeping self-tolerance and immune homeostasis. However, in some cases, they promote tumor progression by inhibiting the effective antitumor response [31]. The low-risk group showed higher level immune infiltrates. Among them, M1 macrophage types are thought to be key factors in antitumorigenesis, production of proinflammatory cytokines, and promotion of T-cell immunity. Furthermore, the study suggested that LAG-3, CTLA4, and TIGIT were highly expressed in the high-risk subgroup and also positively associated with risk score, indicating that the high-risk group is in a more immunosuppressed state by comparison with the low-risk group, but also means that patients of high-risk subgroup may benefit more from immune checkpoint inhibitors. N6-methyladenosine (m6A) RNA methylation plays a crucial role in the tumor immune microenvironment cancer development. A recent study indicates that downregulated m6A-related genes predict unfavorable outcomes in gastric cancer [32]. We assess the association of m6A regulators with risk score, and we found that most of the m6A regulators were significantly decreased in the high-risk subgroup. Autophagy is an essential homeostatic process by which cells decompose their components. Recent studies have uncovered a key role for autophagic pathways and proteins in immunity and inflammation. We thus evaluate the association of autophagy-related genes and the risk score, and results indicate that many autophagy-related genes were significantly correlated with risk scores, particularly the DKK1, SNAI2, and AREG.

## 5. Conclusion

Collectively, our study constructs and validates a robust 9-IFRG risk signature, which may be to the advantage of risk classification and prognosis prediction in KIRC patients. However, there are still some restrictions that should not be overlooked. Our results are mainly derived from bioinformatic analysis; clinical samples and cellular experiments are required to prove our findings; in addition, our analysis discovered that inflammation-related genes might influence renal clear cell carcinoma progression through several mechanisms; nevertheless, further in vivo and in vitro experiments are needed to explore the exact biological roles.

## Figures and Tables

**Table 1:** Univariate Cox regression analysis for identification of prognosis-related IFRGs in the training dataset.

IFRGs, inflammation-related genes				
ID	HR	HR.95L	HR.95H	*P*-value
CD82	1.411	1.154	1.725	7.904*E−*04
SLC4A4	0.770	0.663	0.895	6.653*E−*04
F3	1.298	1.117	1.509	6.529*E−*04
SGMS2	0.627	0.482	0.817	5.291*E−*04
NOD2	1.844	1.316	2.585	3.817*E−*04
TIMP1	1.384	1.161	1.651	2.931*E−*04
RIPK2	1.883	1.340	2.645	2.632*E−*04
BEST1	2.144	1.430	3.216	2.252*E−*04
KLF6	0.683	0.558	0.836	2.213*E−*04
APLNR	0.741	0.634	0.867	1.817*E−*04
ACVR2A	0.405	0.253	0.649	1.742*E−*04
ADRM1	2.553	1.567	4.160	1.677*E−*04
SPHK1	1.639	1.296	2.072	3.751*E−*05
CX3CL1	0.671	0.556	0.811	3.541*E−*05
PDPN	1.545	1.259	1.897	3.089*E−*05
GABBR1	1.690	1.326	2.154	2.226*E−*05
ADORA2B	2.236	1.550	3.227	1.695*E−*05
CALCRL	0.681	0.572	0.810	1.506*E−*05
ROS1	3.007	1.877	4.817	4.677*E−*06
PLAUR	1.812	1.456	2.256	1.032*E−*07

**Table 2:** 9 prognosis-associated hub PRGs identified by multivariate Cox regression analysis.

IFRGs, inflammation-related genes					
ID	coef	HR	HR.95L	HR.95H	*P*-value
ROS1	1.069	2.913	1.533	5.535	1.092*E −* 03
PLAUR	0.339	1.403	1.023	1.924	3.553*E −* 02
ACVR2A	−0.720	0.487	0.272	0.872	1.546*E −* 02
KLF6	−0.198	0.820	0.641	1.050	1.156*E −* 01
GABBR1	0.600	1.822	1.395	2.380	1.073*E −* 05
APLNR	−0.164	0.849	0.713	1.011	6.565*E −* 02
SPHK1	−0.386	0.679	0.467	0.989	4.367*E −* 02
PDPN	0.183	1.200	0.945	1.524	1.337*E −* 01
ADORA2B	0.472	1.603	1.072	2.399	2.165*E −* 02

## Data Availability

The datasets used and/or analyzed during the present study are available from the corresponding author upon reasonable request.
